# T52. N-ACETYL-CYSTEINE ADD-ON TREATMENT LEADS TO AN IMPROVEMENT OF FORNIX WHITE MATTER INTEGRITY IN EARLY PSYCHOSIS

**DOI:** 10.1093/schbul/sby016.328

**Published:** 2018-04-01

**Authors:** Paul Klauser, Lijing Xin, Margot Fournier, Alessandra Griffa, Martine Cleusix, Raoul Jenni, Michel Cuenod, Rolf Gruetter, Patric Hagmann, Philippe Conus, Philipp Baumann, Kim Q Do

**Affiliations:** 1 Lausanne University Hospital; 2 DP-CHUV (TIPP)

## Abstract

**Background:**

Beneficial effects of N-acetyl-cysteine (NAC) on negative symptoms in chronic schizophrenia have been reported in two studies. A recent study in early psychosis from our group, did not report significant improvement in negative symptoms (potentially linked to the modest baseline levels) but showed improvement in cognition (i.e. processing speed) and an increase in the brain antioxidant glutathione (GSH) levels, indicating good target engagement.1 Indeed, research in animal models highlights the critical role of redox regulation by brain GSH for white matter maturation and maintenance. Given the strong evidence of white matter (WM) alterations in schizophrenia as well as the current lack of etiological treatments, redox dysregulation is an interesting target. The current study aims at investigating the impact of NAC, a precursor of GSH, the main antioxidant in the brain, on WM integrity in patients in the early psychosis phase. We focused on the fornix bundle that has been shown to be impaired in an animal model of oxidative stress2 (i.e. impaired GSH synthesis) as well as in early psychosis patients.3

**Methods:**

WM diffusion properties were estimated using generalized fractional anisotropy (gFA) computed from diffusion spectrum imaging (DSI) brain scans acquired in patients who received either NAC (n=10; mean age=25.3 ± 5.7; males/females 9/1) or placebo (n=10; mean age=24.8 ± 7.9; males/females 5/5) as add-on treatment over 6-months. GSH levels were measured in the medial prefrontal cortex using Magnetic Resonance Spectroscopy (MRS).

**Results:**

A non-parametric longitudinal voxel-based analysis limited to the fornix revealed a time x treatment interaction which reached significance in the body of the fornix (corrected p<.04) with NAC patients showing an increase in gFA over 6-month of treatment. Importantly, improvement of gFA (i.e. increase) in the fornix of early psychosis patients (NAC and placebo) correlated with increase in cerebral GSH levels (r=.67; p<.005).

**Discussion:**

This study is the first to assess the effect of NAC on WM integrity as assessed by diffusion weighted-imaging in the early phase of psychosis. WM alterations appear early in the illness and become widespread in a more chronic phase of the disease.4 To the best of our knowledge there is currently no approved medication for schizophrenia that show significant effect on WM integrity. In this study, effects of NAC on WM integrity in the fornix were significant despite the limited sample size. This is a small-scale proof of concept study, which was very demanding for early psychosis patients and needs replication in a larger study. Its potential properties to counteract WM deficits may be even more important in individuals at clinical high risk for psychosis. As NAC add-on treatment is safe with no side effects, this study paves the way for preventive approach at the early stages of psychosis.

**References:**

1. Conus P, Seidman L, Fournier M, et al. N-Acetyl-Cysteine in a double-blind randomized placebo- controlled trial: Towards biomarker guided treatment in early psychosis. Schizophr Bull 2017. DOI:10.1093/schbul/sbx093.

2. Corcoba A, Steullet P, Duarte JMN, et al. Glutathione Deficit Affects the Integrity and Function of the Fimbria/Fornix and Anterior Commissure in Mice: Relevance for Schizophrenia. Int J Neuropsychopharmacol 2015; 19: 1–11.

3. Baumann P, Griffa A, Fournier M, et al. Impaired fornix-hippocampus integrity is linked to peripheral glutathione peroxidase in early psychosis. Transl Psychiatry 2016; 6: 1–8.

4. Klauser P, Baker ST, Cropley VL, et al. White Matter Disruptions in Schizophrenia Are Spatially Widespread and Topologically Converge on Brain Network Hubs. Schizophr Bull 2017; 43: 425–35.

